# A New Labdane-Type Diterpene, 6-*O*-Acetyl-(12*R*)-epiblumdane, from *Stevia rebaudiana* Leaves with Insulin Secretion Effect

**DOI:** 10.3390/biomedicines10040839

**Published:** 2022-04-03

**Authors:** Heesun Kang, Dahae Lee, Ki Sung Kang, Ki Hyun Kim

**Affiliations:** 1 School of Pharmacy, Sungkyunkwan University, Suwon 16419, Korea; hskang428@skku.edu; 2 College of Korean Medicine, Gachon University, Seongnam 13120, Korea; pjsldh@gachon.ac.kr

**Keywords:** *Stevia rebaudiana*, Asteraceae, labdane-type diterpene, insulin secretion

## Abstract

*Stevia rebaudiana* (Asteraceae), commonly known as candyleaf, sweetleaf, or sugarleaf, is a branched bushy shrub whose leaves are used as a natural sweetener owing to the high content of sweet diterpenes. As part of our ongoing work to identify structurally novel and bioactive natural products, phytochemical investigation of the ethanolic extract of *S. rebaudiana* leaves led to the isolation of one new labdane-type diterpene, 6-*O*-acetyl-(12*R*)-epiblumdane (**1**), and nine known terpenoids, including six diterpenes (**2**–**6** and **10**), two monoterpenes (**7** and **8**), and one triterpene (**9**). The structure of the new compound **1** was elucidated via analysis of one- and two-dimensional nuclear magnetic resonance (NMR) spectroscopic data and high-resolution electrospray ionization mass spectrometry data, and its absolute configuration was established using electronic circular dichroism (ECD) calculations and gauge-including atomic orbital NMR chemical shift calculations, followed by DP4 + probability analysis. The isolated compounds **1**–**10** were evaluated for their effects on glucose-stimulated insulin secretion in the INS-1 rat pancreatic β-cell line. The new compound **1**, 6-*O*-acetyl-(12*R*)-epiblumdane, stimulated glucose-stimulated insulin secretion in INS-1 pancreatic β-cells without inducing cytotoxicity. Thus, 6-*O*-acetyl-(12*R*)-epiblumdane (**1**), an active compound derived from *S. rebaudiana* leaves, can be used as a potential therapeutic agent to prevent type 2 diabetes.

## 1. Introduction

*Stevia rebaudiana* Bertoni is a branched bushy shrub of the Asteraceae family, native to the Amambay region in northeast Paraguay. It also grows in neighboring regions of Brazil and Argentina. Its cultivation has recently spread to other regions of the world, including Canada, some parts of Asia, and Europe. *S. rebaudiana* is commonly known as candyleaf, sweetleaf, or sugarleaf, and its leaves are called “Stevia” and used as a natural sweetener due to the high content of sweet diterpene (~4–20%) in the leaf dry matter. The leaves are the source of several *ent*-kaurene diterpenoid glycosides (steviosides), which are responsible for the sweet taste. Among 230 species in the genus *Stevia*, only *S. rebaudiana* and *S. phlebophylla* are known to produce steviosides [1]. Stevia and steviosides have been used as substitutes for saccharose in the treatment of diabetes mellitus, obesity, hypertension, and caries prevention [2]. In addition, as natural sweeteners, steviosides and related compounds may offer health benefits with their anti-hyperglycemic, anti-hypertensive, anti-inflammatory, anti-tumor, anti-diarrheal, diuretic, and immunomodulatory activities [1]. According to previous phytochemical studies, the leaves of *S. rebaudiana* possess diverse bioactive compounds, including flavonoids, alkaloids, chlorophylls, xanthophylls, hydroxycinnamic acids (caffeic acids and chlorogenic acids), oligosaccharides, and amino acids [3]. *S. rebaudiana* leaves are rich in diterpenes, such as sterebins A-N and 6-*O*-acetyl-austroinulin, and diterpenoid glycosides, including stevioside, steviolbioside, rebaudiosides A–F, and dulcoside [4].

As part of an ongoing research project to discover unique bioactive natural products derived from diverse medicinal plants and microbes [5,6,7,8,9,10], the leaves of *S. rebaudiana* were explored for bioactive phytochemicals from their ethanolic (EtOH) extracts. Chemical analysis of the EtOH extract, aided by liquid chromatography–mass spectrometry (LC/MS)-based analysis equipped with an in-house UV spectra library, led to the isolation and identification of a new labdane-type diterpene (**1**) along with nine known compounds (**2**–**10**). The structure of the new labdane-type diterpene (**1**) was characterized by conducting one- and two-dimensional nuclear magnetic resonance (NMR) experiments and high-resolution mass spectrometry (HR-MS), and its absolute configurations were elucidated using electronic circular dichroism (ECD) and gauge-including atomic orbital (GIAO) NMR chemical shift calculations, followed by DP4 + probability analysis. Herein, we describe the separation and structural characterization of the isolated compounds (**1**–**10**), including one new compound, and evaluate their effects on glucose-stimulated insulin secretion (GSIS) in an INS-1 rat pancreatic β-cell line.

## 2. Materials and Methods

### 2.1. General Experimental Procedures

Detailed information on the general experimental procedure is included in the Supplementary Materials.

### 2.2. Plant Material

*S. rebaudiana* leaves were collected in August 2018 from Namyangju-si, Gyeonggi-do, Republic of Korea. The plant material was verified by Prof. K. H. Kim, one of the authors. A voucher specimen, STBA-08-2018, was deposited in the herbarium of the School of Pharmacy affiliated with Sungkyunkwan University, Suwon, Republic of Korea.

### 2.3. Extraction and Isolation

**Figure 1:**
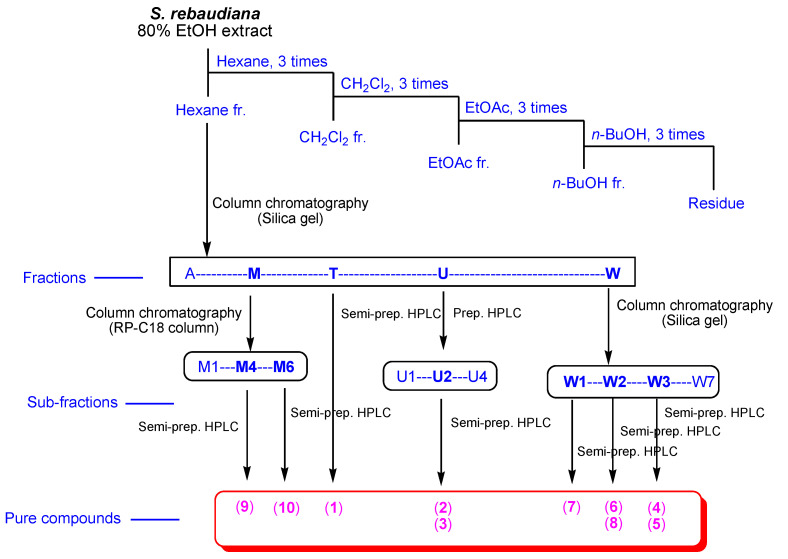
Schematic representation of the isolation process of compounds **1**–**10**.

Dried leaves of *S. rebaudiana* (5 kg) were extracted with 80% aqueous EtOH (each 3.0 L × 3 d) at 25 °C and filtered. The combined filtrate was concentrated under reduced pressure using a rotavapor, which afforded the EtOH extract (169.4 g). Then, the resultant extract was suspended in 700 mL of distilled water and successively solvent-partitioned with hexane (700 mL), dichloromethane (CH_2_Cl_2,_ 700 mL), ethyl acetate (EtOAc, 700 mL), and *n*-butanol (BuOH, 700 mL). Four major fractions with increasing polarity were obtained: hexane-soluble (15.8 g), CH_2_Cl_2_-soluble (3.0 g), EtOAc-soluble (8.9 g), and *n*-BuOH-soluble fractions (12.4 g) (Figure 1). With reference to our in-house UV library database, LC/MS analysis of the four fractions derived from the solvent partitioning revealed the presence of diterpenes without a sugar moiety in the hexane-soluble fraction. Thus, the hexane fraction (15.7 g) was separated over silica gel column chromatography (eluted with hexane/EtOAc (50:1 → 1:1) and 100% MeOH of gradient system) to gain 26 fractions (A–W). Fraction T (70.1 mg) was purified via semi-preparative HPLC (43% MeCN) to yield compound **1** (1.0 mg, *t*_R_ = 55.0 min) (Figure 1). Fraction M (173.1 mg) was subjected to reversed-phase (RP)-C18 column chromatography (eluted with MeOH/H_2_O (70% → 80% → 90% → 100% MeOH) gradient system]) to yield six sub-fractions (M1–M6). Sub-fraction M4 (68.2 mg) was separated using semi-preparative HPLC (MeCN 73%) to yield compound **9** (48.2 mg, *t*_R_ = 55.0 min). Compound **10** (2.0 mg, *t*_R_ = 82.0) was obtained from sub-fraction M6 (16.5 mg) using semi-preparative HPLC (78% MeCN). Four sub-fractions (U1–U4) were obtained from fraction U (167.7 mg) using preparative reversed-phase HPLC with the MeCN/H_2_O (65% → 100% MeCN) gradient system. Sub-fraction U2 (83.2 mg) was purified via semi-preparative HPLC (50% MeCN) to yield compounds **2** (4.4 mg, *t_R_* = 31.0 min) and **3** (4.7 mg, *t_R_* = 50.0 min) (Figure 1). Fraction W (3.4 g) was separated using silica gel chromatography (eluted with CH_2_Cl_2_/MeOH (40:1 → 30:1 → 20:1 → 10:1 → 5:1 → 3:1 → 1:1) and 100% MeOH) gradient system) to yield seven sub-fractions W1–W7. Sub-fraction W1 (238.7 mg) was fractionated using a Sephadex LH-20 column (eluted with a CH_2_Cl_2_/MeOH (2:8) isocratic system) to obtain three sub-fractions (W11–W13). Sub-fraction W13 (146.3 mg) was further fractionated using preparative reverse-phase HPLC with MeCN/H_2_O (30% → 80% MeCN) to yield five sub-fractions (W131–W135). Sub-fraction W132 (24.5 mg) was isolated via semi-preparative HPLC (13% MeCN) to yield compound **7** (0.4 mg, *t*_R_ = 56.0 min). Sub-fraction W2 (128.7 mg) was separated via preparative reversed-phase HPLC with the MeOH/H_2_O (60% → 100% MeOH) gradient system to obtain four sub-fractions (W21–W24). Sub-fraction W21 (9.2 mg) was purified via semi-preparative HPLC (18% MeCN) to obtain compound **8** (0.5 mg, *t_R_* = 38.0). Sub-fraction W22 (14.8 mg) was purified using semi-preparative HPLC (63% MeOH) to yield compound **6** (1.2 mg, *t_R_* = 48.0 min). Sub-fractions W31–W35 were prepared from fraction W3 (388.9 mg) using RP-C18 column chromatography (eluted with MeOH/H_2_O (60% → 70% → 80% → 90% → 100% MeOH) gradient system). Compound **4** (6.5 mg, *t_R_* = 44.8 min) was obtained from sub-fraction W33 (37.0 mg) via semi-preparative HPLC (65% MeOH). Sub-fraction W35 (21.6 mg) was purified using semi-preparative HPLC (65% MeCN) to yield compound **5** (1.0 mg, *t_R_* = 44.0 min). (Figure 1).

#### 6-*O*-Acetyl-(12*R*)-epiblumdane (**1**)

White powder; ${\left[\alpha \right]}_{D}^{25}$ + 27.9 (*c* 0.05, MeOH); UV (MeOH) λ_max_ (log ε) 200 (1.6), 225 (3.6) nm; ECD (MeOH) λ_max_ (Δε) 214 (−29), 253 (−13) nm; ^1^H (850 MHz) and ^13^C (212.5 MHz) NMR data (Table 1); electrospray ionization mass spectrometry (ESI-MS) (positive-ion mode) *m/z* 403.3 [M + Na]^+^; HR-ESI-MS (negative-ion mode) *m/z* 379.2463 [M − H]^−^ (calculated for C_22_H_35_O_5_, 379.2484) and *m/z* 425.2533 [M + HCOO]^−^ (calculated for C_23_H_37_O_7_, 425.2539).

### 2.4. Computational Analysis

All of the proposed conformers were obtained through the MacroModel (version 2019-3, Schrödinger, LLC, New York, NY, USA) module with mixed torsional/low-mode sampling implemented with the MMFF94 force field. All cases of searches were initially set in the gas phase, with a 10 kJ/mol energy window limit and a maximum of 10,000 steps, to explore all potential conformers thoroughly. The Polak–Ribiere conjugate gradient protocol was established with 10,000 maximum iterations and a 0.001 kJ (mol Å)^−1^ convergence threshold on the root mean square gradient to minimize conformers [11,12,13]. The conformers proposed in this study (within 5 kJ/mol found in the MMFF force field) were selected for geometry optimization by TURBOMOLE V7.2, with the density-functional theory settings of B3-LYP/6-31+G(d,p).

Geometrically optimized conformers for the possible diastereomers **1a** and **1b** were used to calculate the GIAO magnetic shielding tensors at the B3-LYP/6-31+G(d,p) level. Chemical shift values were calculated from the magnetic shielding tensors using Equation (1) [14,15,16], where $\delta_{calc}^{x}$ is the calculated NMR chemical shift for nucleus *x* and *σ^o^* is the shielding tensor for the proton and carbon nuclei in tetramethylsilane calculated using the density-functional theory B3-LYP/6-31+G(d,p) basis set.

(1) $$\delta_{calc}^{x}={\;\sigma }^{o}-\sigma^{x}$$

The calculated unscaled NMR properties of the optimized structures were averaged, and the scaled chemical shift values were obtained using Equation (2).

(2) $$\delta_{scaled}=\frac{\delta_{unscaled}-intercept}{slope}$$

The DP4 + probability analysis was performed using an Excel sheet (DP4 +) provided by Grimblat et al. [15].

ECD calculations of the optimized conformers of enantiomers **1a** and **1c** were performed at the B3LYP/6-31+G(d,p) level. The calculated ECD spectra were simulated by overlying each transition, where σ is the width of the band at height 1/e (Equation (3)) and Δ*E*_i_ and *R*_i_ are the excitation and rotatory strengths of transition *i*, respectively. In this study, *σ* was 0.10 eV. The excitation energies and rotational strengths for the ECD spectra were calculated based on the Boltzmann populations of the conformers, and ECD visualization was performed using SigmaPlot 14.0 (Systat Software, Inc., San Jose, CA, USA).

(3) $$\Delta \epsilon \left(E\right)=\frac{1}{2.297\times 10^{-39}}\frac{1}{\sqrt{2\pi \sigma }}\overset{i}{\underset{A}{\sum }}\Delta E_{i}R_{i}e^{[-{\left(E-\Delta E_{i}\right)}^{2}/\left(2\sigma {)}^{2}\right]}$$

### 2.5. Cell Culture

The rat insulin-secreting INS-1 β-cell line was obtained from Biohermes (Shanghai, China) and maintained in an RPMI-1640 medium (Cellgro, Manassas, VA, USA), supplemented with 0.05 mM 2-mercaptoethanol, 11 mM D-glucose, 10 mM N-2-hydroxyethylpiperazine-N-2-ethane sulfonic acid (HEPES), 1% penicillin/streptomycin (Invitrogen Co., Grand Island, NY, USA), 1 mM sodium pyruvate, 2 mM L-glutamine, and 10% fetal bovine serum in a humidified atmosphere containing 5% CO_2_ at 37 °C.

### 2.6. Cell Viability Assay

To measure cell viability, an Ez-Cytox cell viability assay kit purchased from Daeil Lab Service Co. (Seoul, Korea) was used. INS-1 cells were incubated in 96-well plates for 24 h to determine the non-toxic concentration ranges of compounds **1**–**10**. After treatment of the compounds, the Ez-Cytox reagent was added, and absorbance at 450 nm was measured using a microplate reader.

### 2.7. Glucose-Stimulated Insulin Secretion Assay

INS-1 cells were cultured in 12-well plates for 24 h to measure GSIS after treatment with compounds **1**–**10**. After starvation for 2 h, INS-1 cells were treated with the compounds **1**–**10**. After 2 h of incubation, glucose (2.8 and 16.7 mM as basal and stimulant, respectively) was added to each well and incubated further for 1 h. According to the manufacturer’s instructions, GSIS was calculated with the secreted insulin by using a rat insulin ELISA kit (Gentaur, Shibayagi Co. Ltd., Shibukawa, Japan).

### 2.8. Statistical Analysis

Statistical significance was analyzed using one-way analysis of variance (ANOVA) and multiple comparisons with Bonferroni correction. All analyses were carried out using SPSS Statistics ver. 19.0 (SPSS Inc., Chicago, IL, USA), and significant differences were considered present at the 5% level (*p* < 0.05).

## 3. Results and Discussion

### 3.1. Isolation of Compounds ***1***–***10***

**Figure 2:**
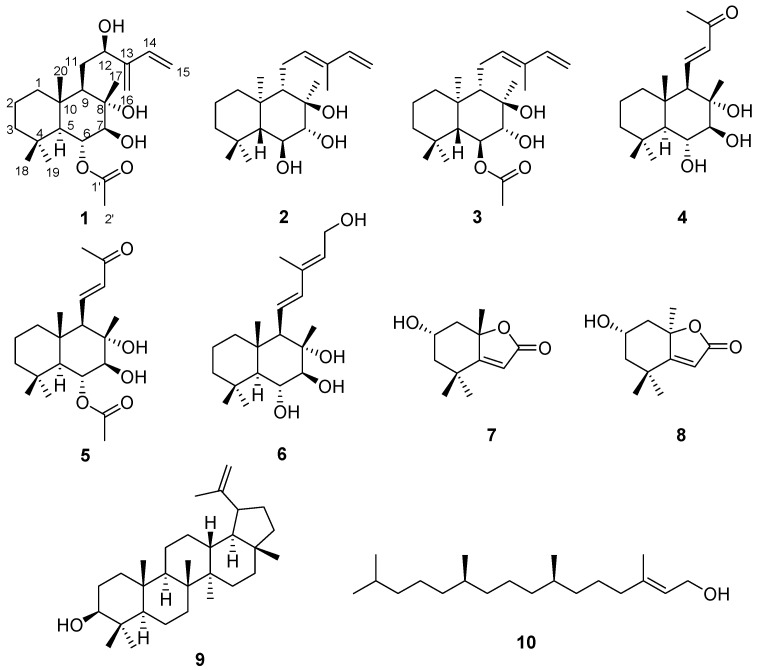
Chemical structures of the isolated compounds **1**–**10**.

Leaves of *S. rebaudiana* were dried and extracted with 80% aqueous EtOH to obtain the crude EtOH extract via rotary evaporation. The resultant EtOH extract was subjected to solvent partitioning using four organic solvents (namely hexane, CH_2_Cl_2_, EtOAc, and *n*-BuOH), which yielded four main fractions. LC/MS-based analysis of the fractions with reference to our in-house UV library suggested that the hexane-soluble fraction is promising for phytochemical investigation because peaks for diterpenes without sugar moieties were detected in the hexane-soluble fraction. Repeated column chromatography and semi-preparative HPLC resulted in the isolation of 10 terpenoids, including seven diterpenes (**1–6** and **10**), two monoterpenes (**7** and **8**), and one triterpene (**9**) (Figure 2) from the hexane-soluble fraction.

### 3.2. Structural Elucidation of the Isolated Compounds

Compound **1**, obtained as a white powder, possessed the molecular formula C_22_H_36_O_5_ (five degrees of unsaturation), as determined by using the negative-ion HR-ESI-MS data at *m*/*z* 379.2463 [M − H]^−^ (calculated for C_22_H_35_O_5_, 379.2484) and *m/z* 425.2533 [M + HCOO]^−^ (calculated for C_23_H_37_O_7_, 425.2539), as well as NMR data (Table 1). The ^1^H NMR data (Table 1) of compound **1** showed the presence of the proton resonances corresponding to five methyl groups (*δ*_H_ 0.88 (3H, s), 0.95 (3H, s), 1.02 (3H, s), 1.28 (3H, s), and 2.15 (3H, s)), three oxygenated methines (*δ*_H_ 3.58 (1H, d, *J* = 10.0 Hz), 4.50 (1H, dd, *J* = 11.0, 2.0 Hz), and 5.14 (1H, dd, *J* = 11.0, 10.0 Hz)), two sets of exo-methylene (*δ*_H_ 5.15 (1H, d, *J* = 11.0 Hz)/5.46 (1H, d, *J* = 17.5 Hz) and 5.30 (1H, s)/5.33 (1H, s)), and an olefinic methine (*δ*_H_ 6.41 (1H, dd, *J* = 17.5, 11.0 Hz)). The ^13^C NMR data (Table 1) of **1**, acquired using heteronuclear single quantum coherence (HSQC) and heteronuclear multiple-bond coherence (HMBC) spectra, showed the presence of 22 carbon resonances, including two olefinic pairs (*δ*_C_ 114.8/136.7 and *δ*_C_ 116.5/145.1), three oxygenated methine carbons (*δ*_C_ 73.1, 83.4, and 83.5), four methylene groups (*δ*_C_ 17.6, 25.9, 39.5, and 43.3), two methine groups (*δ*_C_ 52.9 and 56.5), four non-protonated carbons (*δ*_C_ 33.4, 39.1, 77.8, and 172.1)(including a carbonyl group (*δ*_C_ 172.1)), and five methyl groups (*δ*_C_ 16.6, 19.8, 21.6, 21.8, and 35.7). Comprehensive analysis of the NMR spectral data suggested that the structure of **1** closely resembled that of compound **3**, 6-*O*-acetylaustroinulin [4], which was also isolated as a labdane-type diterpene in this study. The NMR data of **1** were similar to those of **3** except for the additional presence of one hydroxyl group and an exo-methylene unit in **1**. In addition, a literature survey revealed that the NMR data of **1** also have high similarity to those of (12*R*)-epiblumdane [17], except for the presence of an acetyl-moiety in **1**. The gross planar structure of **1** was confirmed by analysis of 2D NMR experiments.

**Figure 3:**
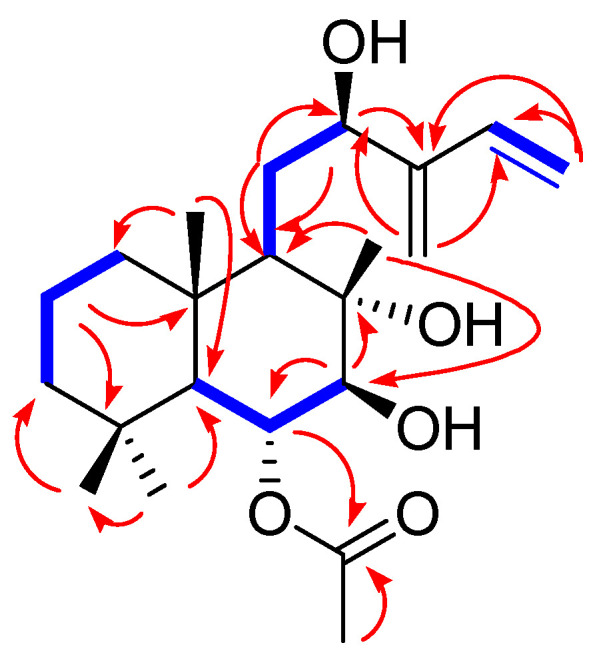
^1^H-^1^H COSY (bold lines) and key HMBC (arrows) correlations of compounds.

The positions of the additional hydroxyl group and exo-methylene units were unambiguously assigned as C-12 and C-13/C-16, respectively, by the HMBC correlations of H_2_-11/C-9, H_2_-11/C-12, H-12/C-9, H-12/C-13 and C-16, H_2_-15/C-13, H_2_-15/C-14, H_2_-16/C-12, H_2_-16/C-13, and H_2_-16/C-14, as well as cross-peaks of H-9/H-11/H-12 and H-14/H-15 in the ^1^H-^1^H correlated spectroscopy (COSY) spectrum (Figure 3), where the partial structure of **1** for the side chain was confirmed as a 2-hydroxy-3-methylene-4-penten-1-yl moiety. In addition, the cross-peaks of H-5/H-6/H-7 in the ^1^H-^1^H COSY spectrum, along with the HMBC correlations of H-6/C-1′ and H-2′/C-1′, were observed, which indicated the presence of an *O*-acetyl-moiety at C-6. The complete interpretation of the ^1^H-^1^H COSY and HMBC data afforded the complete planar structure of **1** (Figure 2).

**Figure 4:**
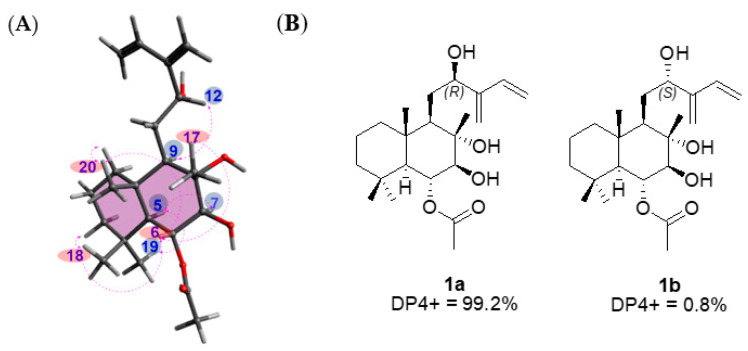
(**A**) Key nuclear Overhauser effect spectroscopy (dashed arrows) correlations of compound **1** and (**B**) DP4 + analysis and probability scores for compound **1** with **1a**/**1b**.

**Figure 5:**
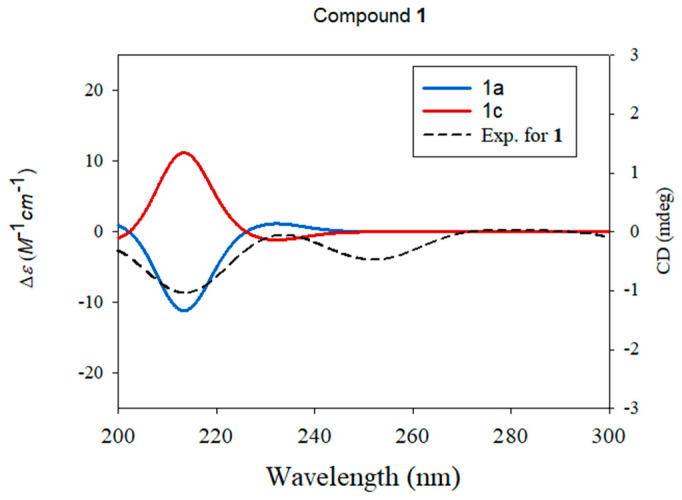
Experimental and calculated ECD spectra of compound **1**.

The relative configuration of compound **1** was established using nuclear Overhauser effect spectroscopy, which showed the correlations of H-5/H_3_-19, H-5/H-7, H-6/H_3_-17, H-6/H_3_-18, H-6/H_3_-20, and H-9/H-12 (Figure 4). Based on this result, the relative configurations of **1** were unambiguously determined to be 5S*, 6R*, 7S*, 8S*, 9R*, and 10S*. To determine the stereochemistry of C-12, an additional chemical reaction was required; however, Mosher’s reaction was not an option owing to the limited substance of **1** because compound **1** was isolated in only 1.0 mg. To verify the stereochemistry of C-12 in **1**, GIAO NMR chemical shift calculations were performed, followed by DP4 + analysis. The computationally calculated ^1^H and ^13^C NMR chemical shifts of two possible diastereomers **1a** and **1b** were compared with the experimental values of **1** by utilizing DP4 + probability analysis, which revealed the structural equivalence of **1** to **1a** with 99.2% probability (Figure 4B). Finally, to verify the absolute configuration of **1**, two possible isomers, **1a** (5*S*, 6*R*, 7*S*, 8*S*, 9*R*, 10*S*, and 12*R*) and **1c** (5*R*, 6*S*, 7*R*, 8*R*, 9*S*, 10*R*, and 12*S*), were used for ECD calculations, and the quantum chemically calculated ECD data were compared with the experimental ECD spectrum of **1**. The experimental ECD spectrum of **1** was in agreement with that of **1a** (Figure 5), which indicates the absolute configuration of **1** as 5*S*, 6*R*, 7*S*, 8*S*, 9*R*, 10*S*, and 12*R*. Thus, the chemical structure of compound **1** was elucidated (Figure 2), and the compound was named 6-*O*-acetyl-(12*R*)-epiblumdane.

The isolated known compounds were identified as austroinlin (**2**) [4], 6-*O*-acetylaustroinulin (**3**) [4], sterebin A (**4**) [17], sterebin B (**5**) [18], sterebin E (**6**) [19], (+)-epiloliolide (**7**) [20], (−)-loliolide (**8**) [21], lupeol (**9**) [22], and phytol (**10**) [23] using LC/MS analysis and the comparison of their spectroscopic data, including the ^1^H and ^13^C NMR spectra, and physical data with previously reported values.

### 3.3. Effect of Compounds on Glucose-Stimulated Insulin Secretion

**Figure 6:**
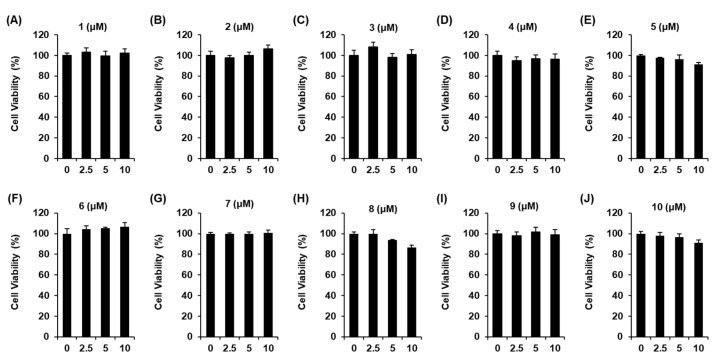
Effect of compounds on the viability of INS-1 cells. Effect of compounds **1**–**10** (**A**–**J**) on the viability of INS-1 cells compared to the control (0 µM) (*n* = 3 for independent experiments), as assessed using MTT assay for 24 h.

*S. rebaudiana* leaves have been proven to be effective against diabetes [24,25]. However, few experimental studies have been performed to identify the antidiabetic compounds from *S. rebaudiana* leaf extracts. It’s well-known that the progression of type 2 diabetes is characterized by the defects in glucose-stimulated insulin secretion (GSIS) [26]. Hence, we investigated the effects of compounds **1**–**10** on GSIS in an INS-1 rat pancreatic β cell line. To select non-toxic concentrations of compounds **1**–**10** for performing the GSIS assay, their effects on cell viability were assessed using the Ez-Cytox cell viability assay. Compounds **1**–**10** did not show any toxicity at concentrations ranging from 2.5 to 10 μM (Figure 6). 

**Figure 7:**
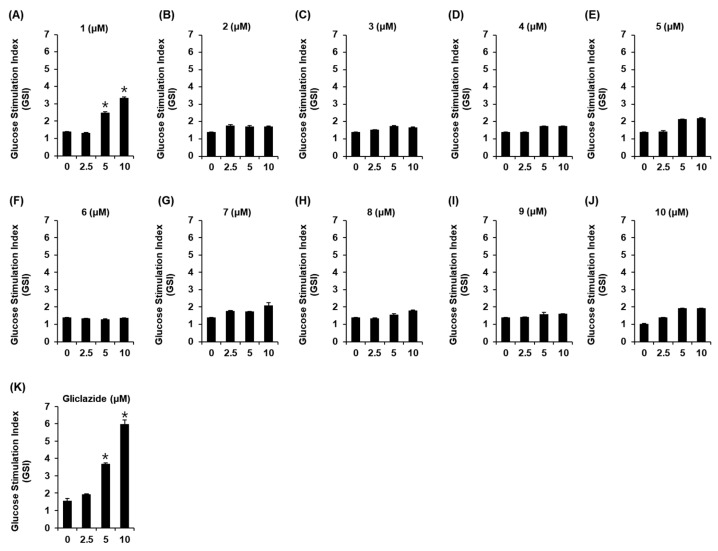
Effect of compounds on glucose-stimulated insulin secretion (GSIS) in INS-1 cells. Effect of compounds **1**–**10** (**A**–**J**) and gliclazide (positive control) (**K**) on GSIS expressed as the glucose stimulation index (GSI) in INS-1 cells compared with the control (0 μM). GSI = insulin concentration at 16.7 mM (high) glucose and insulin concentration at 3.3 mM (low) glucose. (*n* = 3 independent experiments, * *p* < 0.05, Kruskal–Wallis nonparametric test). Data represent the mean ± standard error of the mean (SEM).

Thus, the highest concentration of all the compounds was set to 10 µM in the GSIS assay. To determine the efficacy, compounds **1**–**10** were screened in INS-1 rat pancreatic β cells. Among these compounds, the new compound **1** significantly increased GSIS (Figure 7). The GSI values of compound **1** were 2.51 ± 0.04 and 3.34 ± 0.05 at concentrations of 5 and 10 μM, respectively.

## 4. Conclusions

In this study, chemical investigation of the extracts of *S. rebaudiana* leaves resulted in the isolation of one new labdane-type diterpene, 6-*O*-acetyl-(12*R*)-epiblumdane (**1**), and nine known terpenoids, including six diterpenes (**2–6** and **10**), two monoterpenes (**7** and **8**), and one triterpene (**9**). The structure of the new compound **1** was elucidated by analyzing one- and two-dimensional NMR data, HR-ESI-MS data, and NMR chemical shift calculations, followed by DP4 + probability analysis as well as quantum chemical ECD calculations. We found that 6-*O*-acetyl-(12*R*)-epiblumdane (**1**) increased glucose-stimulated insulin secretion in an INS-1 rat pancreatic β-cell line. Our results suggest that 6-*O*-acetyl-(12*R*)-epiblumdane (**1**), an active compound derived from *S. rebaudiana* leaves, can be applied as a potential antidiabetic agent. Further studies will be required to elucidate the exact mechanism by which the active compound **1** prevents type 2 diabetes.

## Figures and Tables

**Table 1:** ^1^H (850 MHz) and ^13^C NMR (212.5 MHz) data of compound **1** in CDCl_3_ (δ ppm) ^a^.

Position	Compound 1	
	*δ*_H_ (*J* in Hz)	*δ*_C_, Multiplicity
1	1.20 m/1.44 m	39.5 t
2	1.45 m/1.58 m	17.6 t
3	1.24 m ^b^/1.37 m ^b^	43.3 t
4		33.4 s
5	1.47 m ^b^	56.5 d
6	5.14 dd (11.0, 10.0)	73.1 d
7	3.58 d (10.0)	83.5 d
8		77.8 d
9	1.81 d (4.0)	52.9 d
10		39.1 s
11	1.56 m ^b^, 2.13 m ^b^	25.9 t
12	4.50 dd (11.0, 2.0)	83.4 d
13		145.1 s
14	6.41 dd (17.5, 11.0)	136.7 d
15	5.15 d (11.0)/5.46 d (17.5)	114.8 t
16	5.30 s/5.33 s	116.5 t
17	1.28 s	19.8 q
18	0.88 s	21.8 q
19	1.02 s	35.7 q
20	0.95 s	16.6 q
1’		172.1 s
2’	2.15 s	21.6 q

^a^ *J* values are in Hz and are shown in parentheses. ^13^C NMR assignments are based on HMBC experiments. ^b^ Signals partially obscured.

## Data Availability

Not applicable.

## Supplementary Materials

The following are available online at https://www.mdpi.com/article/10.3390/biomedicines10040839/s1. Figure S1: HR-ESI-MS of compound **1**; Figure S2: UV spectrum of compound **1**; Figure S3: ^1^H NMR spectrum of compound **1**; Figure S4: ^1^H−^1^H COSY spectrum of compound **1**; Figure S5: heteronuclear single quantum coherence (HSQC) spectrum of compound **1**; Figure S6: HMBC spectrum of compound **1**; Figure S7: Nuclear Overhauser effect spectrum of compound **1**; Figure S8: DP4+ probability analysis of compound **1** using an Excel sheet; NMR and physical data of the isolated compounds **2**–**10**.
