# Supplementary material for: A New Labdane-Type Diterpene, 6-O-Acetyl-(12R)-epiblumdane, from Stevia rebaudiana Leaves with Insulin Secretion Effect
Source: Biomedicines. 2022 Apr 3;10(4):839. doi: 10.3390/biomedicines10040839 (PMC9026343; doi:10.3390/biomedicines10040839)
Supplement: Supplementary file 1 [file biomedicines-10-00839-s001.zip › biomedicines-1624732-supplementary.pdf]

Supplementary Materials

A New Labdane-type Diterpene, 6-*O*-Acetyl-(12*R*)-epiblumdane from *Stevia rebaudiana* Leaves with Insulin Secretion Effect

Heesun Kang [1,†], Dahae Lee [2,†], Ki Sung Kang [2,*] and Ki Hyun Kim [1,*]

[1] School of Pharmacy, Sungkyunkwan University, Suwon 16419, Korea; hskang428@skku.edu (H.K.)
[2] College of Korean Medicine, Gachon University, Seongnam 13120, Korea; pjsldh@gachon.ac.kr (D.L.)
† These authors contributed equally to this work
* Correspondence: kkang@gachon.ac.kr (K.S.K.); khkim83@skku.edu (K.H.K.); Tel.: +82-31-750-5402 (K.S.K.); +82-31-290-7700 (K.H.K.)

Figure S1 : HR-ESI-MS of compound **1**
Figure S2 : UV spectrum of compound **1**
Figure S3 : ^{1}H NMR spectrum of compound **1**
Figure S4 : ^{1}H–^{1}H COSY spectrum of compound **1**
Figure S5 : HSQC spectrum of compound **1**
Figure S6 : HMBC spectrum of compound **1**
Figure S7 : NOESY spectrum of compound **1**
Figure S8 : DP4+ probability analysis of compound **1** using an Excel sheet

NMR and physical data of the isolated compounds 2-10

General experimental procedures

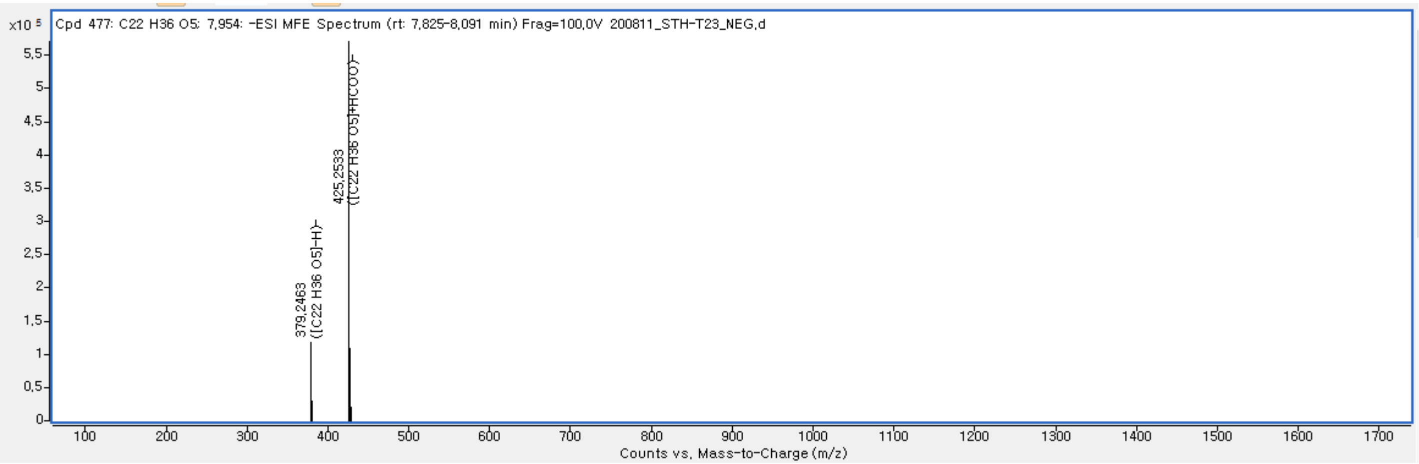

Cpd. 477: C22 H36 O5

Name	Formula	RT	RI	Mass	CAS	ID Source	Score	Algorithm	Lib/DB
	C22 H36 O5	7.954		380.2548		MFG	82.36	MFE	

Species	m/z	Score (Lib)	Num Spectra	Score (DB)	Hits	Score (MFG)	Score (RT)
(M-H)- (M+HCOO)-	379.2463 425.2533				2	82.36	

Figure S1 : HR-ESI-MS of compound **1**

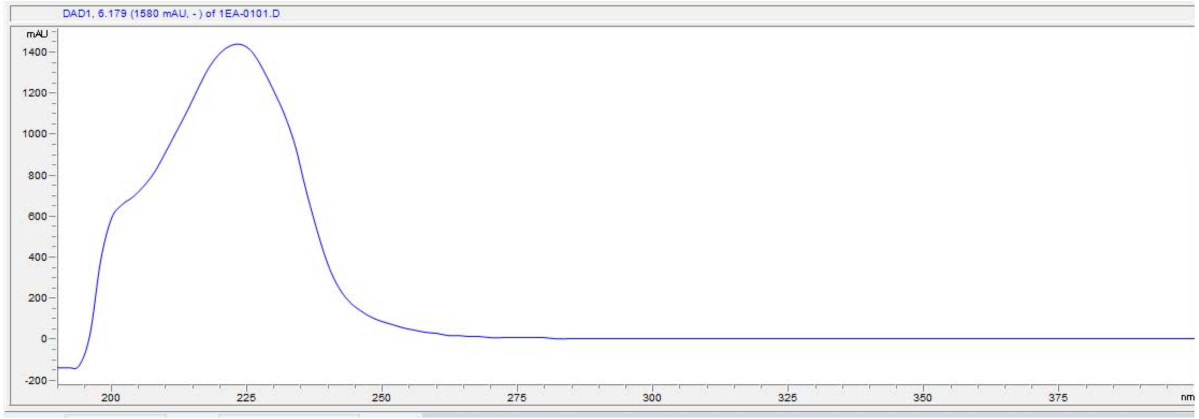

Figure S2 : UV spectrum of compound **1**

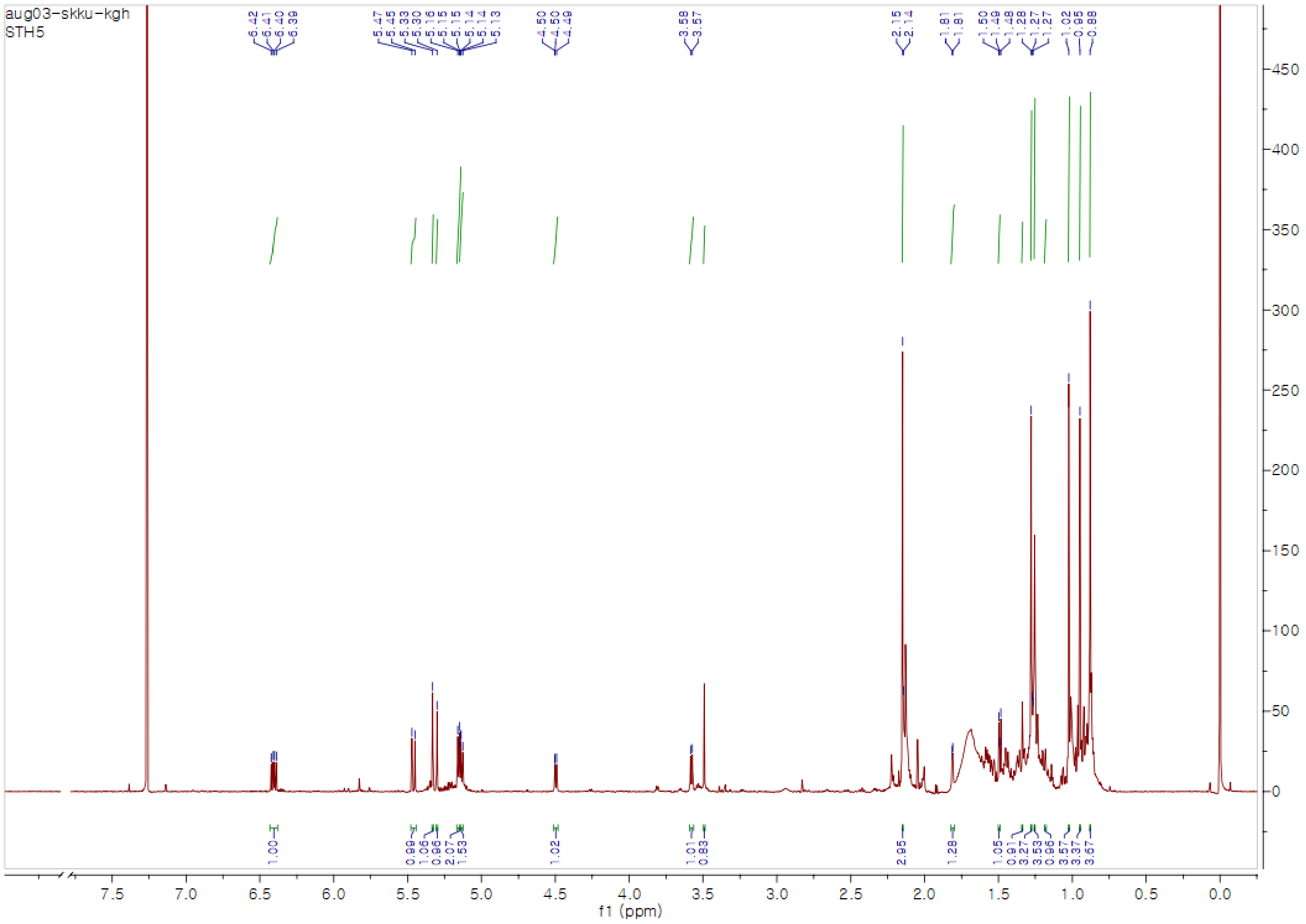

Figure S3 : ^{1}H NMR spectrum of compound **1**

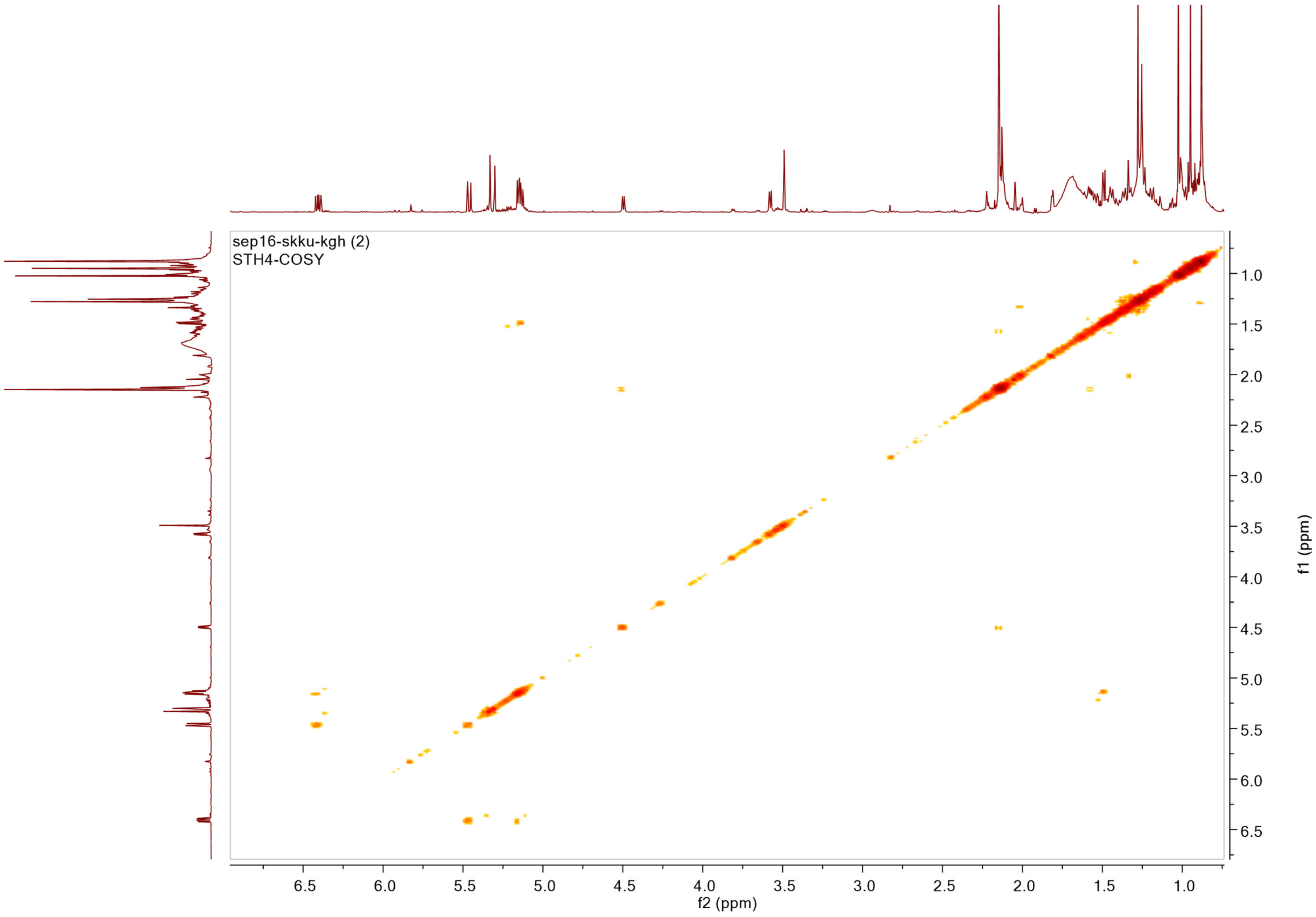

Figure S4 : $^{1}H-^{1}H$ COSY spectrum of compound **1**

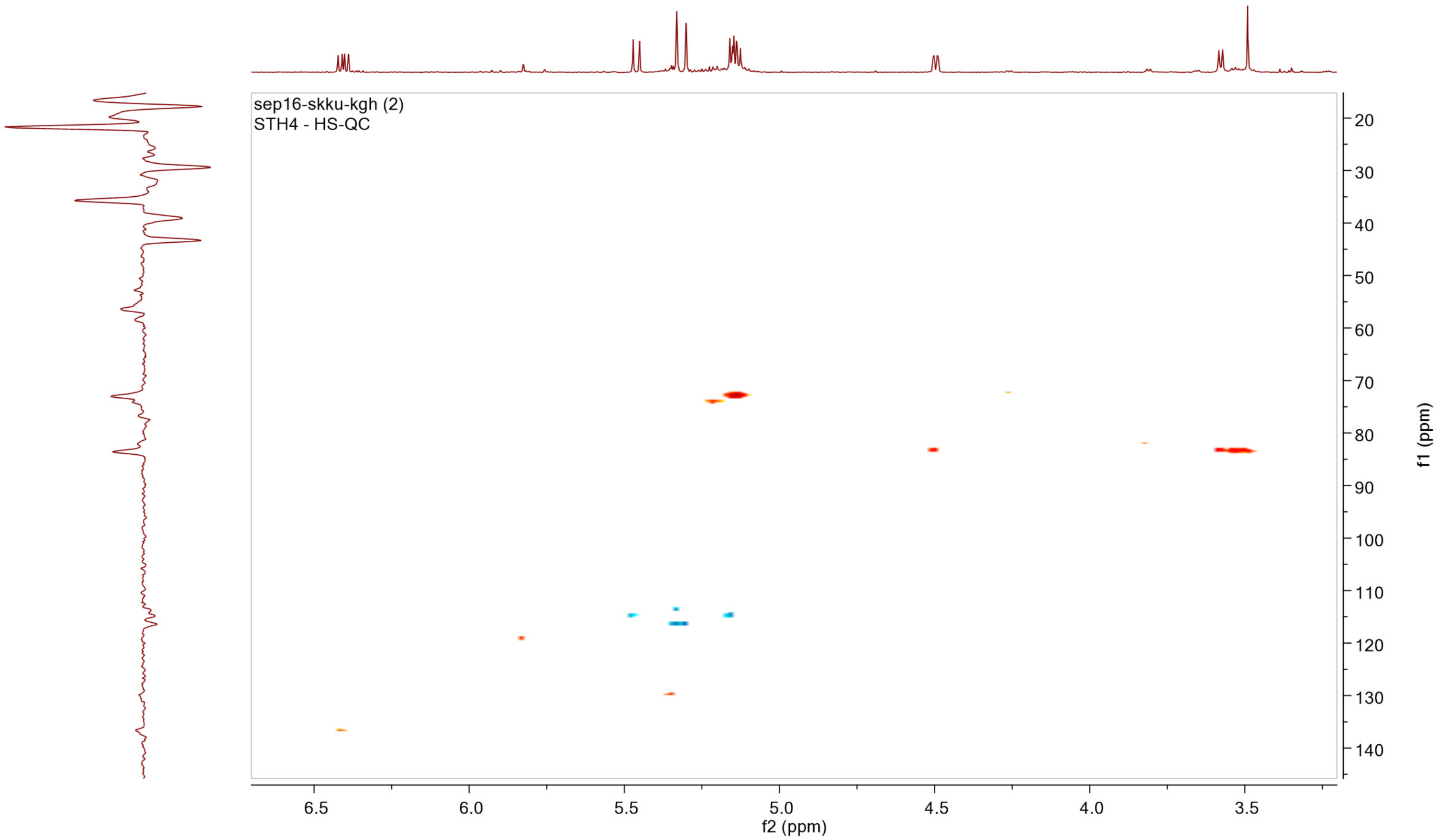
sep16-skku-kgh (2)
STH4 - HS-QC
f2 (ppm)
f1 (ppm)
6.5
6.0
5.5
5.0
4.5
4.0
3.5
20
30
40
50
60
70
80
90
100
110
120
130
140

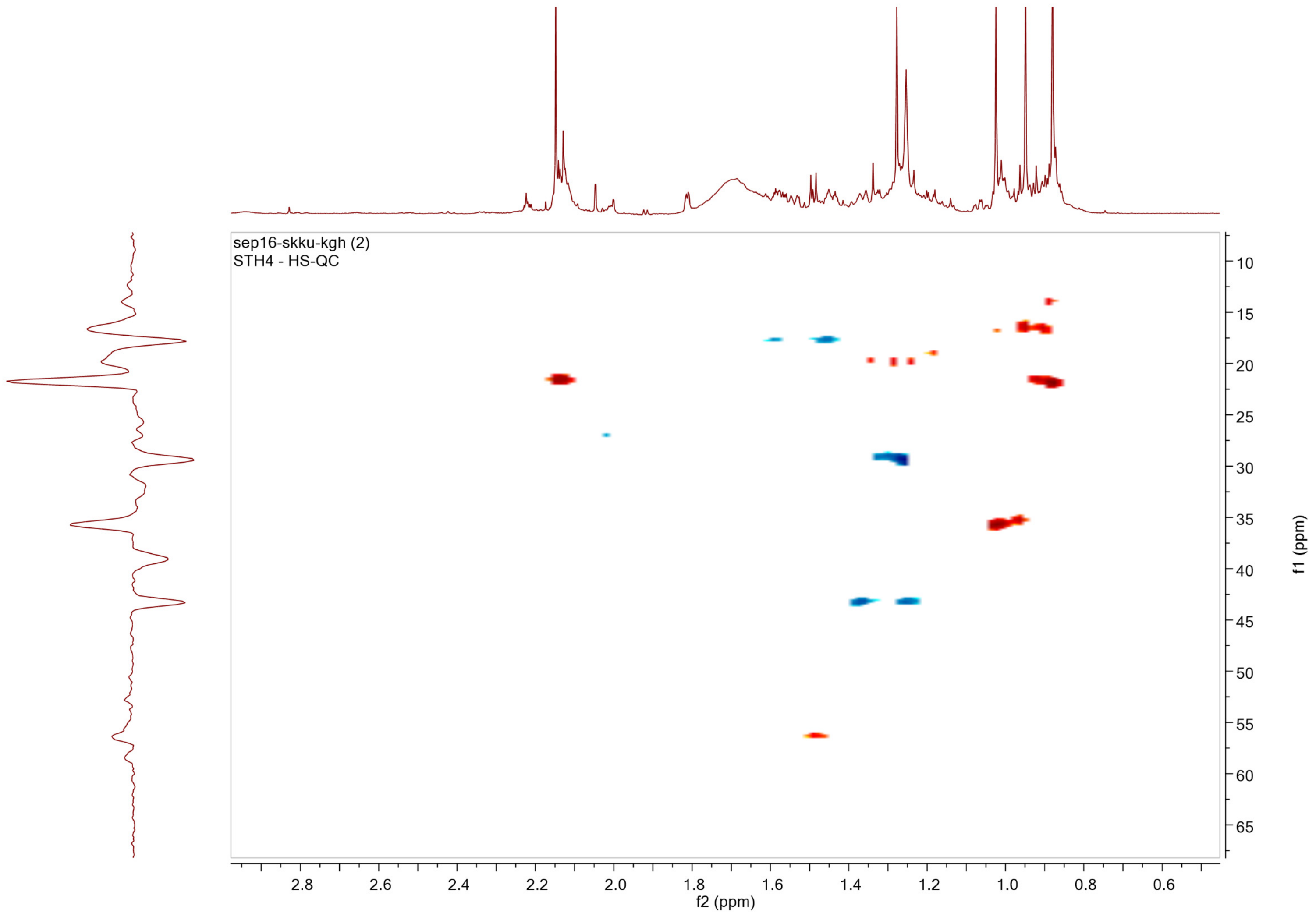

Figure S5 : HSQC spectrum of compound **1**

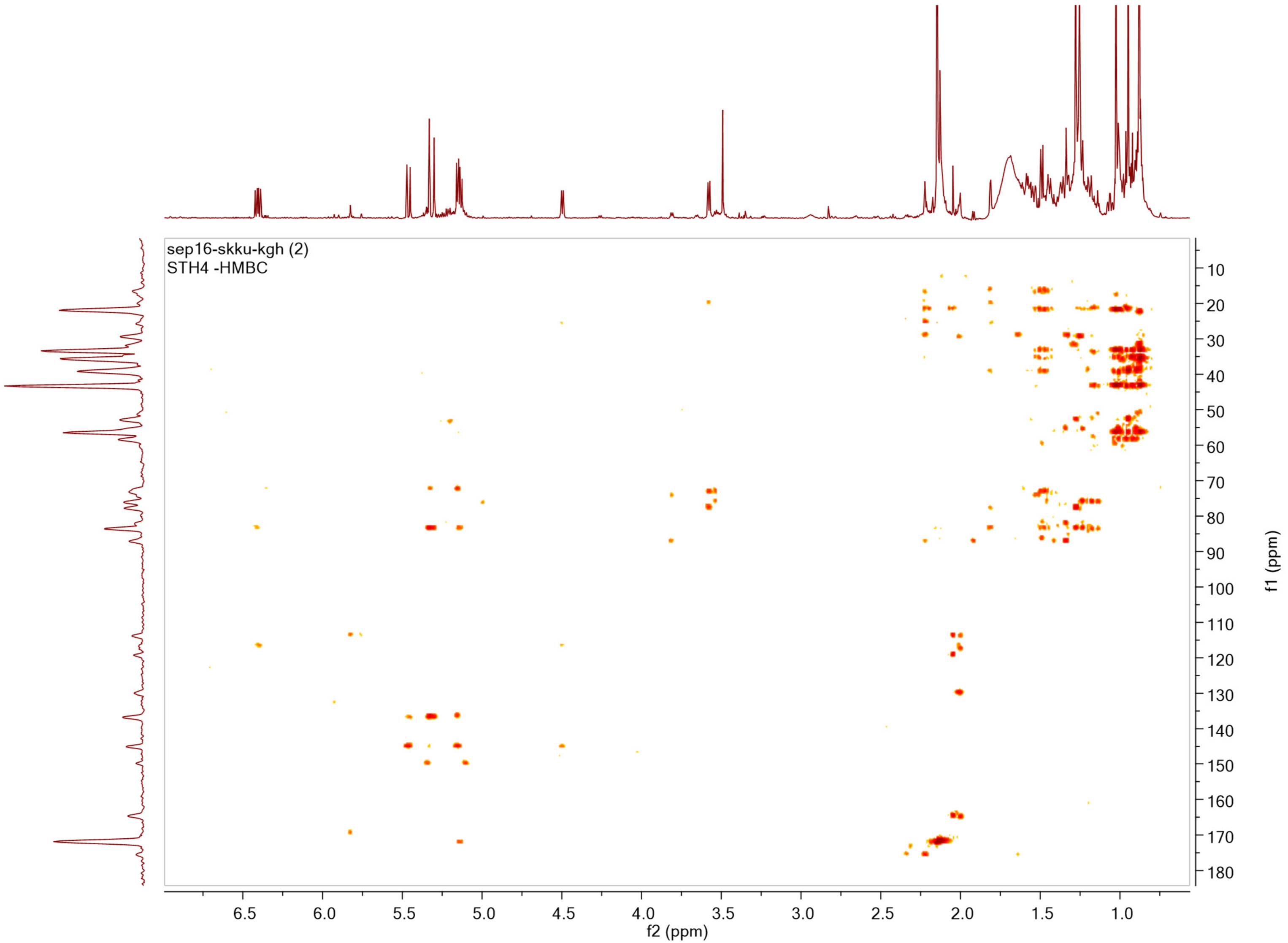

Figure S6 : HMBC spectrum of compound **1**

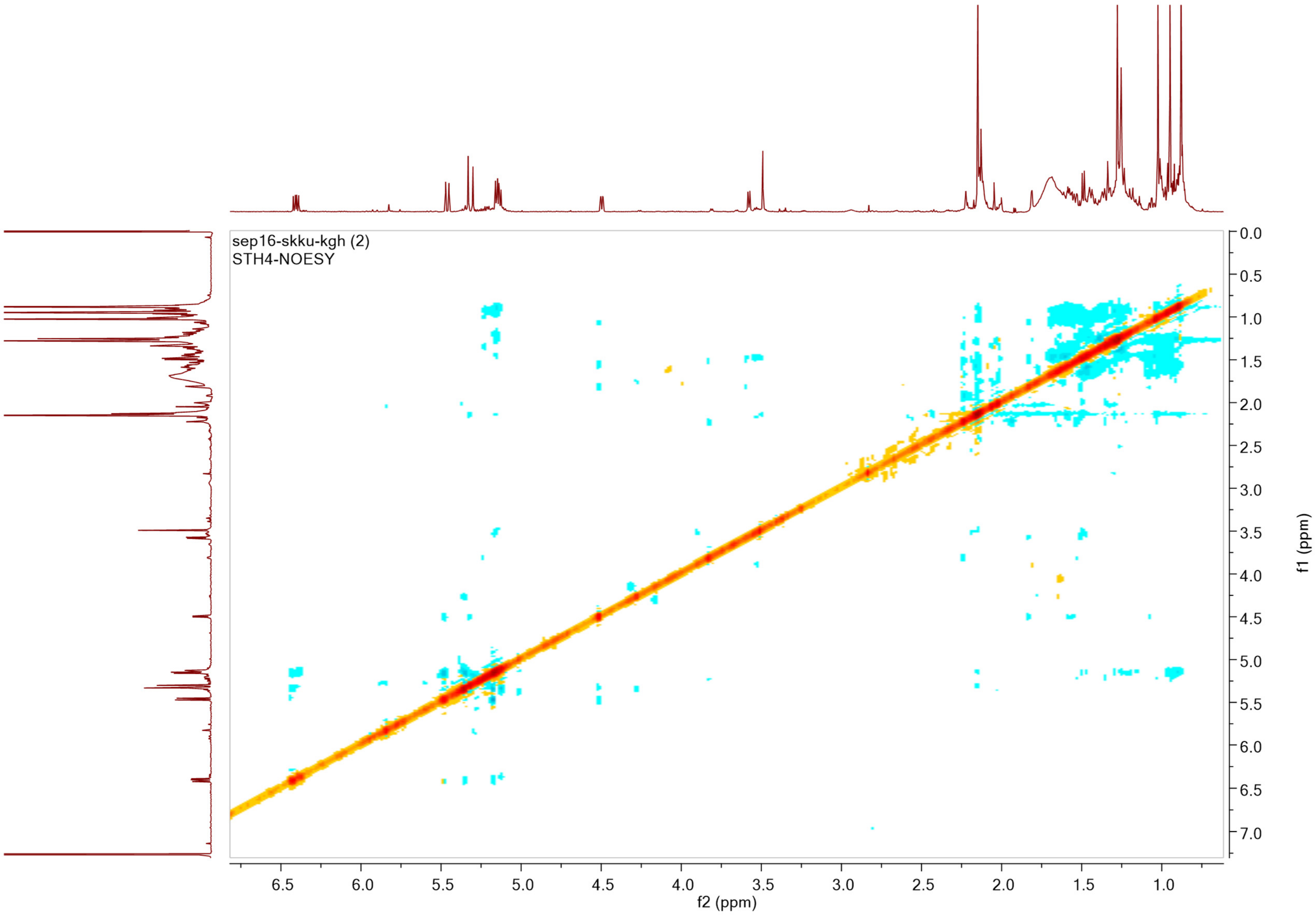

Figure S7 : NOESY spectrum of compound **1**

Functional	Solvent?		Basis Set		Type of Data	
B3LYP	PCM		6-31G(d,p)		Unscaled Shifts	
	Isomer 1	Isomer 2	Isomer 3	Isomer 4	Isomer 5	Isomer 6
sDP4+ (H data)	1.09%	98.91%	-	-	-	-
sDP4+ (C data)	99.14%	0.86%	-	-	-	-
sDP4+ (all data)	55.98%	44.02%	-	-	-	-
uDP4+ (H data)	98.21%	1.79%	-	-	-	-
uDP4+ (C data)	64.14%	35.86%	-	-	-	-
uDP4+ (all data)	98.99%	1.01%	-	-	-	-
DP4+ (H data)	37.76%	62.24%	-	-	-	-
DP4+ (C data)	99.52%	0.48%	-	-	-	-
DP4+ (all data)	99.20%	0.80%	-	-	-	-

Figure S8 : DP4+ probability analysis of compound **1** using an Excel sheet

NMR and physical data of the isolated compounds 2-10

1. Austroinlin (**2**)

White crystals; ^{1}H NMR (850 MHz, $CDCl_3$): *δ* 0.91 (3H, s, H-18), 1.00 (3H, s, H-20), 1.17 (3H, s, H-17), 1.18 (3H, s, H-19), 1.11–1.51 (6H, m, H-1,2,3,5,9), 1.79 (3H, s, H-16), 2.22 (1H, m, H-11a), 2.46 (1H, m, H-11b), 3.38 (1H, t, *J* = 9.3 Hz, H-7), 3.62 (1H, t, *J* = 10.1 Hz, H-6), 5.11(1H, d, *J* = 10.8 Hz, H-15), 5.21 (1H, d, *J* = 17.2 Hz, H-15), 5.44(1H, t, *J* = 6.9 Hz, H-12), 6.86(1H, dd, *J* = 17.2 and 10.8 Hz, H-14); ESI-MS *m/z* 345.3 $[M+Na]^+$.

2. 6-*O*-Acetylaustroinulin (**3**)

White crystals; ^{1}H NMR (850 MHz, $CDCl_3$): *δ* 0.88 (3H, s, H-18), 0.96 (3H, s, H-20), 1.01 (3H, s, H-19), 1.23 (3H, s, H-17), 1.79 (3H, s, H-16), 2.13 (3H, s, H-22), 2.23 (1H, dd, *J* = 15.3 and 7.0, H-11a), 2.48 (1H, d, *J* = 15.7 Hz, H-11b), 3.47 (1H, d, *J* = 10.0 Hz, H-7), 5.11(1H, d, *J* = 10.7 Hz, H-6), 5.13 (1H, d, *J* = 10.6 Hz, H-15), 5.20 (1H, d, *J* = 17.2 Hz, H-15) 5.44(1H, t, *J* = 6.8 Hz, H-12), 6.87 (1H, dd, *J* = 17.2 and 10.8 Hz, H-14); ESI-MS *m/z* 387.3 $[M+Na]^+$.

3. Sterebin A (**4**)

Colorless powder; ^{1}H NMR (850 MHz, $CDCl_3$): *δ* 1.04 (3H, s, H-17), 1.07 (3H, s, H-18), 1.18 (3H, s, H-16), 1.26 (3H, s, H-15), 2.02 (1H, d, *J* = 10.4 Hz, H-9), 2.28 (3H, s, H-14), 3.44 (1H, d, *J* = 9.4 Hz, H-7), 3.74 (1H, dd, J = 10.7 and 9.7 Hz H-6), 6.22 (1H, d, *J* = 15.5 Hz, H-12), 6.81 (1H, dd, *J* = 15.5, 10.4 Hz, H-11); ESI-MS *m/z* 333.2 $[M+Na]^+$.

4. Sterebin B (**5**)

Colorless powder; ^{1}H NMR (850 MHz, $CDCl_3$): *δ* 0.91 (3H, s, H-17), 1.03 (3H, s, H-18), 1.12 (3H, s, H-16), 1.31 (3H, s, H-15), 1.44 (1H, d, *J* = 11.0 Hz,) 2.04 (1H, d, J = 10.3 Hz, H-9), 2.14 (3H, s, H-14), 2.28 (3H, s, H-2') 3.53 (1H, d, *J* = 9.9 Hz, H-7), 5.22 (1H, dd, *J* = 11.3, 10.0 Hz, H-6), 6.23 (1H, d, *J* = 15.5 Hz, H-12), 6.80 (1H, dd, *J* = 15.5, 10.3 Hz, H-11); ESI-MS *m/z* 375.2 $[M+Na]^+$.

5. Sterebin E (**6**)

Colorless powder; ^{1}H NMR (850 MHz, $CDCl_3$): *δ* 1.01 (3H, s, H-18), 1.03 (3H, s, H-19), 1.18 (3H, s, H-20), 1.21 (3H, s, H-17), 1.83 (3H, s, H-16) 1.88 (1H, d, *J* = 10.1 Hz, H-9) 3.46 (1H, d, *J* = 9.4 Hz, H-7), 3.74 (1H, t, *J* = 10.1 Hz, H-6), 4.29 (2H, d, *J* = 6.8 Hz, H-15) 5.65 (1H, t, *J* = 7.6 Hz, H-11), 5.66 (1H, dd, *J* = 16.0, 10.0 Hz, H-14), 6.19 (1H, d, *J* = 15.4 Hz, H-12); ESI-MS *m/z* 361.2 $[M+Na]^+$.

6. (+)-Epiloliolide (**7**)

Colorless gum; ^{1}H NMR (850 MHz, $CDCl_3$): *δ* 1.28 (3H, s, H-10), 1.31 (3H, s, H-9), 1.34 (1H, dd, J = 12.0 Hz, H-8), 1.51 (1H, t, *J* = 11.8, H-7), 1.59 (3H, s, H-8), 2.04 (1H, ddd, *J* = 12.8, 4.2, and 2.2 Hz, H-5), 2.54 (ddd, *J* = 11.8, 3.9, and 2.3 Hz, H-7), 4.13 (1H, t, *J* = 10.2 Hz, H-6), 5.72 (1H, s, H-3); ESI-MS *m/z* .197.1 $[M+H]^+$.

7. (-)-Loliolide (**8**)

Colorless oil; ^{1}H NMR (850 MHz, $CDCl_3$): 1.26 (3H, s, H-9), 1.47(3H, s, H-8), 1.54 (1H, dd, *J* = 14.7, 3.7 Hz, H-7), 1.77 (1H, m, H-5), 1.78 (3H, s, H-10), 1.97 (1H, dt, *J* = 14.5, 2.6 Hz, H-7), 2.46 (1H, dt, *J* = 14.1 and 2.6 Hz, H-5), 4.34(1H, d, J = 2.5 Hz, H-6), 5.70 (1H, s, H-3); ESI-MS *m/z* .197.1 $[M+H]^+$.

8. Lupeol (**9**)

White powder; ^{1}H NMR (850 MHz, $CDCl_3$): *δ* 0.76, 0.79, 0.83, 0.94, 0.97, 1.03, and 1.68 (each 3H, s, H-23, H-24, H-25, H-26, H-27, H-28, and H-30), 2.38 (1H, td, *J* = 11.1, 5.9 Hz, H-19), 3.19 (1H, dd, *J* = 11.7, 4.6 Hz, H-3), 4.57 (1H, s, H-29b), 4.69 (1H, d, *J* = 2.2, H-29a); ESIMS *m/z*: 449.3 $[M + Na]^+$.

9. Phytol (**10**)

Colorless oil; ^{1}H NMR (850 MHz, $CDCl_3$): *δ* 0.84 (3H, d, *J* = 6.3 Hz , H-19), 0.85 (3H, d, *J* = 6.2 Hz, H-18), 0.86 (3H, d, *J* = 1.4 , H-17), 0.87 (3H, d, *J* = 1.4 , H-16), 1.52 (1H, dt, *J* = 13.3 and 6.7, H-15) 1.67 (3H, s, H-20), 4.15 (2H, d, *J* = 6.9 Hz, H-1), 5.41 (1H, tdd, *J* = 6.9, 2.4, 1.2 Hz, H-2); ESIMS *m/z*: 319.3 $[M + Na]^+$.

General experimental procedures

Optical rotation was measured using a Jasco P-2000 polarimeter (Jasco, Easton, MD, USA). ECD spectra were obtained using a Jasco J-1500 spectropolarimeter (Jasco). Ultraviolet (UV) spectra were acquired using an Agilent 8453 UV-visible spectrophotometer (Agilent Technologies, Santa Clara, CA, USA). NMR spectra were recorded using a Bruker AVANCE III HD 850 NMR spectrometer with a 5 mm TCI CryoProbe operating at 850 MHz (^{1}H) and 212.5 MHz (^{13}C), with chemical shifts given in ppm (*δ*) for ^{1}H and ^{13}C NMR analyses. Preparative and semi-preparative high-performance liquid chromatography (HPLC) was performed using a Waters 1525 Binary HPLC pump with a Waters 996 photodiode array detector (Waters Corporation, Milford, MA, USA) using an Agilent Eclipse C18 column (250 × 21.2 mm, 5 µm; flow rate: 5 mL/min; Agilent Technologies) and a Phenomenex Luna Phenyl-hexyl 100 Å column (250 × 10 mm, 5 µm; flow rate: 2 mL/min; Phenomenex, Torrance, CA, USA). Liquid chromatography/mass spectrometry (LC/MS) analysis was performed on an Agilent 1200 Series HPLC system equipped with a diode array detector and 6130 Series ESI mass spectrometer using an analytical Kinetex C_{18} 100 Å column (100 × 2.1 mm, 5 µm; flow rate: 0.3 mL/min; Phenomenex). All high resolution electrospray ionization mass spectrometry (HR-ESI-MS) data were obtained using an Agilent G6545B quadrupole time-of-flight mass spectrometer (Agilent Technologies) with an Agilent EclipsePlus C_{18} column (2.1 mm × 50 mm i.d., 1.8 µm; flow rate: 0.3 mL/min) maintained at 25 °C. Silica gel 60 (230–400 mesh; Merck, Darmstadt, Germany) and RP-C_{18} silica gel (230–400 mesh; Merck) were used for column chromatography. Molecular sieve column chromatography was performed using a Sephadex LH-20 (Pharmacia, Uppsala, Sweden). Thin-layer chromatography was performed using precoated silica gel F_{254} plates and RP-C_{18} F_{254s} plates (Merck), and the spots were detected under UV light or by heating after spraying with anisaldehyde-sulfuric acid. The three-dimensional molecular modeling was performed using ChemBioDraw Ultra and Avogadro.
